# HER-2 Evaluation in a Specific Gastric Cancer Population with the Highest Rate of Mortality in Spain

**DOI:** 10.1155/2011/391564

**Published:** 2011-10-27

**Authors:** E. U. Cidon, R. G. Centeno, E. G. Lagarto, J. I. Peral

**Affiliations:** ^1^Oncology Department, Clinical University Hospital, 47005 Valladolid, Spain; ^2^Pathology Department, Clinical University Hospital, 47005 Valladolid, Spain

## Abstract

Gastric cancer (GC) still represents the second cause of cancer-related death worldwide. Radical resection is the mainstay of early stages treatment with little impact on overall survival (OS) in the advanced ones. HER-2 is the most relevant biological factor involved. *Purpose*. This study aims to show the relationship between HER-2 positivity and survival in patients with completely resected GC. *Methods*. Retrospective study of GC patients diagnosed in 2003–2005 at our institution. Surgical specimens underwent immunohistochemistry (IHC), and in cases +/++/+++ samples underwent also fluorescence in situ hybridisation (FISH) analyses of HER-2 and graduated according to experts' consensus. *Results*. 120 cases included. Overall expression detected in 7.5%. Correlation between HER-2 positive and female sex, advanced stages or histological grades, or intestinal type was detected. Early recurrences higher in HER-2 positive (66.6% versus 35.4%, *P* = 0.048). The median DFS for c-erbB-2 positive was 15 months (range 2–67 months), and OS was 25 months (range 10–67 months). In the case of patients with c-erbB-2, negative median DFS was 27 months (range 5–67 months) and OS for this sample is 47 months (range 29–67 months). *Conclusions*. These results emphasize the relevance of HER-2 positivity in GC as independent prognostic factor and support its current analyses in daily practice.

## 1. Introduction

Despite the overall incidence of gastric cancer (GC) has declined in recent decades, it still represents the second cause of cancer-related death worldwide [1]. There is a wide geographical variation in both patterns of incidence and survival rates [2]. The highest rates of survival have been recorded in Japan, where its high incidence has led to implement screening measures among the population at high risk. These measures have achieved higher rates of earlier detection with an excellent prognosis [3–5].

Spain records a survival rate above the European average, with 23% of patients alive at 5 years, except in one of its areas with higher incidence rates (Castilla and León) where it has been registered the highest mortality [6]. 

Nowadays, the radical resection is still the mainstay of treatment and despite the progress made in surgical techniques and development of new therapeutic strategies with chemotherapy or perioperative chemoradiotherapy, there have not been a significant improvement in the overall survival (OS) [3, 7]. 

In the recent years, there has been a change in the pattern of presentation of GC [7], with a relative increase in the incidence of proximal tumors. Despite differences in their biological behavior with a more aggressive clinical course than distal tumours, little progress has been made in the knowledge of new prognostic factors. Thus, TNM stage is still the most relevant prognostic factor. Therefore, there is a wide heterogeneity among individuals with the same tumor stage. At this time, it would be necessary to have additional classification parameters or biomarkers to identify subgroups of patients with different biological profiles that correlate more closely with a prognosis and/or response to treatment [8]. 

In this context, a better understanding of the biological basis of GC would be helpful. Recently, many biological factors involved in the genesis of sporadic GC have been known, although their clinical relevance has not been confirmed for most of them [9]. HER-2 protein is a transmembrane tyrosine kinase receptor and a member of the epidermal growth factor receptors (EGFRs) family [8]. The family comprises four members (HER-1 to HER-4). All except HER-3 share the same molecular structure with an extracellular domain of ligand binding, a short transmembrane domain, and an intracellular domain with tyrosine kinase activity. HER-3 is a distinctive member of this family because its kinase domain lacks certain residues that are known to be essential for catalytic activity in other kinases [10].

Ligand binding to its receptor triggers a cascade of signals that influence many aspects of tumor biology as important as cell differentiation, proliferation, and cell survival [11, 12]. 

HER-2 protein is encoded by a gene located on chromosome 17q21 that acts as oncogene in carcinomas because its overexpression induces amplification of the protein in the cell membrane, and consequently the malignant cell gets favorable for their growth properties. 

The role of HER-2 in the development of many types of cancer is well known, especially in invasive breast cancer in which overexpression of the protein in the cell membrane and/or gene amplification has been detected in 10–34%, correlating with worse prognosis and being a predictor of poor response to chemotherapy and hormonal treatments [8]. Although this overexpression has also been detected in other tumors, including GC, its prognostic impact has not been definitively established [13, 14]. The interest in this biological factor has been increased by the presence of trastuzumab, a humanized IgG1 monoclonal antibody that specifically binds the extracellular domain of HER-2 and increases survival rates in breast tumors early and at advanced stage [8]. These results encourage to investigate the prognostic significance of HER-2 protein in other types of tumors with high incidence and few therapeutic options, such as GC. Overexpression of HER-2 in this disease was first described in 1986 by immunohistochemistry (IHC) [8]. Several published series have shown positivity rates varying between 6.8% and 34% for IHC and 7.1% to 42.6% for the amplification of gene copy number by fluorescence in situ hybridization or FISH [15–17]. 

They have also shown higher positivity rates in intestinal type tumors and proximal location (gastroesophageal junction) [8]. 

On the other hand, Funato et al., using GC cell lines that overexpress HER-2, found that this conferred sensitivity to cisplatin, suggesting that positivity could be a predictor of response to this drug [18]. 

Although several studies have attempted to demonstrate its prognostic role, this has not been definitively proven [13, 14]. Some studies have suggested that overexpression and/or amplification in GC after radical resection would be an independent prognostic factor related to the depth of gastric wall invasion, lymph node involvement, undifferentiated tumours, and reduced disease-free survival (DFS) or OS [15–17, 19]. 

This study tries to demonstrate the relationship between HER-2 positivity and survival in a specific population of patients living in an area of high risk of GC in the center of Spain who underwent a complete resection. 

## 2. Material and Methods

### 2.1. Study Design and Eligible Population

This was designed as a retrospective study of patients diagnosed with GC in the period from 2003 to 2005 which were analysed histopathologically at the Department of Pathology of the Clinical University Hospital of Valladolid (Castilla and León, Spain). Men and women older than 18 years of age were eligible for inclusion if they had histologically confirmed adenocarcinoma of the stomach or gastrooesophageal junction. All of them had to be undergone a complete radical resection R0 and followed our institutional standard followup. Major exclusion criteria included induction chemotherapy or metastases at diagnosis. 

### 2.2. Pathological Study

The analyses were performed in tissue sections of 3 mm tissue blocks conventionally fixed in formalin and embedded in paraffin. These samples were subjected to analysis of overexpression by IHC, and in cases with +/++/+++ these samples underwent also an analysis of HER-2 amplification using the techniques described below. 

Tumours were tested for HER-2 status with IHC through HercepTest kit (DAKO K5207 manual process number of the sample) according to manufacturer protocols. For the assessment by IHC, we used the membrane stain graduations on a scale of 4 grades: 0, 1+, 2+, 3+ according to the recommendations of the panel scoring GC [20] (see Table 1). 

We performed also FISH. For this procedure, we used the DAKO kit pharmDx. The rate of HER-2 amplification was determined by the ratio between the number of copies of HER-2/number of centromeres (ratio of HER-2/CEP 17 (centromeric probe 17)). A rate of ≥2 was considered positive, indicating gene amplification. Polysomy of chromosome 17 was defined as ≥3 CEP 17 signals per cell on average. 

The analysis was carried out by two experienced pathologists independently. If no consistent assessment, the case was analyzed by both of them, and the result was obtained by consensus.

This technique was used in conjunction with IHC routinely in all patients with at least +IHC to assess concordance as it has been explained above.

### 2.3. Endpoints

The primary endpoints were DFS and OS. DFS was defined as time from radical surgery until recurrence, death of any cause other than cancer, or last followup. OS was considered of radical resection until death from any cause.

Secondary endpoints included frequency of overexpression and/or amplification in GC, time to progression, and correlation between the status of the overexpression and/or amplification with other pathological, anatomic, or relevant demographic features. 

### 2.4. Study Variables

We reviewed the medical records of each case and collected the clinical, demographic and pathological data. 

Demographic data were area of origin of patients, age, and sex. 

Clinical and pathological data are, for example, tumor location, endoscopic appearance, histologic type of Lauren, endoscopic diagnosis date, date of radical resection, TNM pathologic stage, ratio of metastatic nodes/analyzed nodes, adjuvant treatment, date of recurrence, location of recurrence, relapse type (single/multiple), date of withdrawal or last follow-up visit schedule, patient status at the last visit, date of death, and cause. 

### 2.5. Statistical Analysis

The data were included in a database designed for this purpose for subsequent statistical analysis using SPSS version 8.0. 

We conducted a descriptive study of case characteristics in a pooled analysis stratified according to HER-2 status. 

The statistical association has been evaluated using the chi-square test. 

Quantitative variables were expressed as median and range. Qualitative variables in absolute numbers and percentages. The survival data were analyzed according to Kaplan-Meier method, and statistical significance was tested using log-rank test. We have performed univariate and multivariate analysis to identify the role of overexpression and/or amplification of HER-2 and other factors as prognostic indicators independently associated with DFS and OS. 

## 3. Results

### 3.1. Positive Rate for c-erbB-2 and Correlation with Other Clinicopathologic Factors

We analysed 120 cases of patients underwent a complete radical resection for GC. Patient characteristics are described in Table 2.

High expression of HER-2 protein by IHC 3+ was recorded in 8 cases, and overall expression (IHC 3+ or IHC 2+ and FISH positive) was detected in 7.5% of tumours. These results are described in Table 3. 

The incidence of *c-erbB-2* positive was higher in women with tumors located primarily antropyloric (although at this point has not been detected statistical significance) and intestinal type. Also a greater proportion of patients detected with advanced tumors (pT3-4 and nodes involved) in the subgroup of *c-erbB-2* positive, as shown in Table 4. 

### 3.2. Recurrence

After a median followup of 38 months (range 15–67 months), in patients with HER-2 positive 66.6% recurrences were recorded (6 patients out of 9). 

In these cases, the median time to recurrence was 9 months (range 2–16 months) compared to 19 months (range 15–38 months) in the sample of patients with HER-2 negative, these differences being statistically significant (*P* = 0.04). 

Most of recurrences in HER-2 positive tumours were at distance or combined (locoregional and distant), while in 1 of them only locoregional recurrence was detected in a control endoscopy although he was unfit to radical treatment. Among patients who had recurrence of their tumour, it was observed a statistically significant difference in time to recurrence according to the status of *c-erbB-2*. Thus, the proportion of patients falling on the first year (early relapse) after diagnosis was higher in the HER-2 positive subgroup (4 out of 6 patients) than in the negative one (66.6% versus 35.4%, *P* = 0.048).

### 3.3. Survival

Throughout the study period, we documented two (22.3%) deaths due to tumor disease in the subgroup of patients with *c-erbB-2* positive. In this subgroup, there were three people alive without evidence of disease. The rest are undergoing palliative treatment. The median DFS for the population *c-erbB-2* positive was 15 months (range 2–67 months) meanwhile for HER-2 negative was 27 months (range 5–67 months) (*P* = 0.04). OS for HER-2 positive was 25 months (range 10–67 months) but with *c-erbB-2* negative was 47 months (range 29–67 months) (*P* = 0.039).

### 3.4. Univariate and Multivariate Analysis

Our univariate analysis showed that the status of HER-2, sex, advanced stage, and lymph node metastases were associated with poor survival. 

A multivariate Cox analysis only identified the status of *c-erbB-2* and the advanced stage as independent prognostic factors (data not included).

## 4. Discussion

Our results have found that overexpression of the protein HER-2 by IHC confirmed by amplification of the HER-2/neu oncogene in cases of IHC ++, and are independent prognostic factors in GC after radical resection, correlated statistically significant with early relapse (during a year after radical resection). 

In this regard, we found that, among recurrences that appear in this time period, 66.6% were in the HER-2 positive subgroup compared to 35.4% detected in the *c-erbB-2* negative (*P* = 0.048). 

On the other hand, the univariate and multivariate analyses also revealed that it is an independent factor that correlates with worse survival. Both facts make our results consistent with other published studies, prospective or retrospective, suggesting that the routine assessment of HER-2/neu gene status should be a rule as it is a new prognostic factor in GC.

This analysis detected also a statistically significant difference in the median time to relapse according to HER-2 status subgroups. In this way we have obtained a shorter time in patients with HER-2 positive (median 9 months) compared to HER-2 negative sample (19 months). Similarly there are differences in DFS or OS favoring those cases in which overexpression and/or amplification of *c-erbB-2* was not detected. 

The study by García et al., published in Ann of Surg Oncol 2003, revealed that survival data were also favorable to patients with HER-2 negative in a statistically significant way [19] as our study found. 

We have also identified a statistically significant association between positivity for *c-erbB-2* and female sex, undifferentiated histological-grade tumours, intestinal histological type, and presence of nodal metastases, showing an interesting correlation with the presence of more than 6 nodes metastatic (*P* = 0.052) although with a marginal significance. 

On the contrary we have not detected significant association with tumor location (antropyloric versus other locations) although most were located in antropylorus. In this subject we differ from other published studies which have found a significant correlation between positivity for *c-erbB-2* and the presence of tumours at the gastroesophageal junction. Tanner et al. found that positive rates ranged from 24% to 12% for tumours located at the union or in other areas of the stomach, respectively, [21]. The ToGA trial revealed in the same way and got a range between 32% and 18% [22], respectively. 

On the other hand, the concordance of protein overexpression and gene amplification in GC has been controversial. Recent studies have reported a high correlation between both overexpression by IHC and amplification by FISH. 

The study by Yano et al. found an overall concordance rate of 87% (58.8% for IHC ++ and 88% in cases of IHC +++) [23]. And ToGA trial has also found high rates of overall concordance (87%) [22]. 

All these studies have also showed high rates of agreement between surgical specimens and biopsy specimens. 

Against the others, our study has obtained lower concordance rates, much different than those described above. The overall agreement we have obtained was 42.8%, which means that only 9 cases of 21 positive by IHC (+ to +++) showed oncogene amplification by FISH. In the literature it is thought to be due to cases with IHC 0/1+ which had also gene amplification by FISH. In ToGA trial this fact was detected in almost the same percentage of IHC ++ and FISH positive (23% and 26%, resp.) [22]. But we did not tested by FISH those cases with IHC 0. 

We have identified a concordance of 100% in cases with tumours HER-2 with +++ by IHC so that all patients had oncogene amplification by FISH. By contrast, in cases of IHC ++ concordance was much lower, only found in 16.6% of patients. 

This finding could support the hypothesis that the amplification of the oncogene *c-erbB-2* may not be the only mechanism involved in the overexpression of the protein in the cell membrane in GC as suggested Kameda et al. [24]. On the other hand, the work of Hollywood and Hurst [25] also suggested that overexpression of HER-2 may occur through other molecular strategies such as transcriptional activation by other genes. Another plausible explanation for this discrepancy could be related to the fact of low HER-2 overexpression in CG. This may involve a higher rate of false positives to the nonspecific binding of primary antibody or to the overvaluation of the union. In summary, our study showed a low positivity rate of HER-2 but similar to other series published in the literature, approaching to the initial series published in the 1990s or even the latest Grávalos and Jimeno study [8]. This is probably due to the exclusion of patients with metastases at diagnosis or even the extended period of time considered for the inclusion of patients in our project, with many differences in the protocols of fixation of the specimens that might contribute somewhat to the appearance of false negatives.

## 5. Conclusions

The CG is the second leading cause of cancer death worldwide because most are diagnosed in advanced stages with no chance of cure. In this context, the systemic chemotherapy remains the only treatment option, although with poor results, so new strategies are needed. 

Every time there is more evidence of the importance of HER-2 in these patients, with the positivity correlated with poor survival outcomes due to the increased aggressiveness of the disease. 

There are experimental models showing that trastuzumab (a monoclonal antibody that inhibits HER-2) suppresses the growth of human GC. As a result of these promising data, there have been conducted clinical trials of this antibody in combination with platinum-based chemotherapy with good results. 

Our work contributes to emphasize the importance of this biomarker in the context of a high-risk GC population in the center of Spain. Although this is a retrospective study with the limitations already described, the results provide us with a better understanding of the relevance of this protein as a prognostic marker and indirectly as a therapeutic target and encourage us to continue researching in molecular pathways to improve the overall results and to optimize the clinical application. 

## Figures and Tables

**Table 1:** Immunohistochemistry scoring for HER2 in gastric and gastrooesophageal junction cancer, by type of diagnostic specimen.

IHC	Surgical specimen	Biopsy specimen	HER2 overexpression interpretation
0	No reactivity or membranous reactivity in <10% of tumour cells	No reactivity or no membranous reactivity in any tumour cell	Negative

+	Faint or barely perceptible membranous reactivity in ≥10% of tumour cells; cells are reactive only in part of their membrane	Tumour cell cluster with a faint or barely perceptible membranous reactivity irrespective of percentage of tumour cells stained	Negative

++	Weak to moderate complete, basolateral, or lateral membranous reactivity in ≥10% of tumour cells	Tumour cell cluster with a weak to moderate complete, basolateral, or lateral membranous reactivity irrespective of percentage of tumour cells stained	Equivocal

+++	Strong complete, basolateral, or lateral membranous reactivity in ≥10% of tumour cells	Tumour cell cluster with a strong complete, basolateral, or lateral membranous reactivity irrespective of percentage of tumour cells stained	Positive

**Table 2:** Characteristics of the population studied.

Characteristics	*N*
Median age (range)	56 (26–83)
Sex	
Men	72
Women	48
Location	
Antrum-pylorus	94
Gastrooesophageal and corpus	26
Type of gastric cancer	
Intestinal	59
Diffuse/mixed	25/46
Nodes	
Involved	98
Not involved	22

**Table 3:** HER-2 positivity.

IHC	*N*	FISH	Concordance rate
**+++**	8	8 amplifications	100%
**++**	6	1 amplifications	16,6%
**+**	7	0	0%

**Table 4:** HER-2 positive and clinicopathological factors correlation.

Clinicopathological factor	*N*	*P*
Sex		
Male	3	0.019
Female	6	
Histological grade		
1	3	0.024
2	2	
3	4	
Histological type		
Intestinal	8	0.038
Other	1	
Location		
Antral-pylorus	7	0.096
Oesophagogastric union and corpus	2	
pT		
pT3-pT4	7	0.034
Other	2	
Metastatic nodes		
Yes	7	0.031
No	2	
